# COVID-19 in Pediatric Patients With Acute Lymphoblastic Leukemia or Lymphoma

**DOI:** 10.1001/jamanetworkopen.2023.55727

**Published:** 2024-02-16

**Authors:** Saman K. Hashmi, Jessica Bodea, Tushar Patni, Savannah Angel, Nickhill H. Bhakta, Sima Jeha, Seth E. Karol, Raul C. Ribeiro, Jeffrey E. Rubnitz, Joshua Wolf, Yimei Li, Ching-Hon Pui, Diego R. Hijano, Hiroto Inaba

**Affiliations:** 1 Department of Oncology, St Jude Children’s Research Hospital, Memphis, Tennessee; 2 Department of Global Pediatric Medicine, St Jude Children’s Research Hospital, Memphis, Tennessee; 3 Department of Biostatistics, St Jude Children’s Research Hospital, Memphis, Tennessee; 4 Department of Infectious Diseases, St Jude Children’s Research Hospital, Memphis, Tennessee

## Abstract

**Importance:**

COVID-19 in pediatric patients with acute lymphoblastic leukemia or lymphoma (ALL/LLy) has not been described in detail and may affect chemotherapy administration and long-term outcomes.

**Objective:**

To describe the clinical presentation of COVID-19 and chemotherapy modifications in pediatric patients with ALL/LLy.

**Design, Setting, and Participants:**

This is a retrospective case series of patients at St Jude Children’s Research Hospital and its affiliate sites with newly diagnosed ALL/LLy who were treated on the Total XVII protocol (NCT03117751) between March 30, 2020, and June 20, 2022. Participants included patients aged 1 to 18 years who were receiving protocol chemotherapy. Acute symptoms and chemotherapy modifications were evaluated for 60 days after the COVID-19 diagnosis, and viral clearance, adverse events, and second SARS-CoV-2 infections were followed up during the 27-month study period.

**Exposures:**

SARS-CoV-2; all patients were screened at least weekly and at symptom onset and/or after known exposure to SARS-CoV-2.

**Main Outcomes and Measures:**

Description of the spectrum of COVID-19 illness and chemotherapy modifications.

**Results:**

Of 308 pediatric patients, 110 (36%) developed COVID-19 at a median age of 8.2 (IQR, 5.3-14.5) years. Sixty-eight patients (62%) were male. Most patients were in the continuation/maintenance phase of chemotherapy (101 [92%]). Severe disease was rare (7 [6%]) but was associated with older age, higher white blood cell counts at ALL/LLy diagnosis, lower absolute lymphocyte counts at COVID-19 diagnosis, abnormal chest imaging findings, and SARS-CoV-2 reinfection. Rare but serious thrombotic events included pulmonary embolism and cerebral venous sinus thrombosis (n = 1 for each). No multisystem inflammatory syndrome in children or death was seen. SARS-CoV-2 reinfection occurred in 11 patients (10%) and was associated with older age and with receiving standard or high-risk vs low-risk ALL/LLy therapy. Chemotherapy interruptions occurred in 96 patients (87%) and were longer for patients with severe disease, SARS-CoV-2 reinfection, and/or a COVID-19 diagnosis during the pre-Omicron variant period vs the post-Omicron period (after December 27, 2021).

**Conclusions and Relevance:**

In this case series of COVID-19 in pediatric patients with ALL/LLy, severe COVID-19 was rare, but chemotherapy administration was affected in most patients. Long-term studies are needed to establish the outcomes of COVID-19 in this population.

## Key Points

**Question:**

What is the spectrum of COVID-19 and associated chemotherapy modifications in pediatric patients with acute lymphoblastic leukemia/lymphoma (ALL/LLy)?

**Findings:**

In this case series of 308 patients, 110 developed COVID-19 while receiving ALL/LLy therapy: only 7 of these patients had severe illness, and 11 had a second episode of COVID-19. Chemotherapy interruptions occurred in 96 patients and were longer in patients with severe illness.

**Meaning:**

The findings of this study suggest that although severe COVID-19 was rare, most patients experienced chemotherapy interruptions regardless of COVID-19 severity, which may affect survival outcomes for patients with ALL/LLy.

## Teaser

This case series examines the outcomes regarding COVID-19 severity and chemotherapy alterations in children with acute lymphoblastic leukemia or lymphoma.

## Introduction

The clinical presentation of COVID-19 ranges from an absence of symptoms to severe respiratory distress and multisystem organ failure.^1^ The symptoms appear milder in children than in adults, although severe pediatric cases have been reported.^2,3,4^ In children with cancer, the 30-day mortality rate is less than 4%, compared with 24% to 31% in adults.^2,5^ Pediatric mortality has been higher in low- to middle-income countries than in high-income countries (4.3% vs 0.4%).^6^ Other risk factors for mortality in pediatric populations have included older age, low absolute lymphocyte and neutrophil counts, and intensive treatment regimens for cancer.^2,6^ Chemotherapy varies based on the diagnosis, and most studies of COVID-19 in pediatric cancer have been heterogeneous, combining different diagnoses and patients with newly diagnosed and relapsed disease.^7,8,9,10^ Detailed reports of COVID-19 in the context of specific cancer diagnoses are scarce.

Herein, we present the clinical course of COVID-19 in pediatric patients with newly diagnosed acute lymphoblastic leukemia (ALL) or lymphoblastic lymphoma (LLy), the most common pediatric cancers, who were treated on a single treatment protocol at St Jude Children’s Research Hospital (St Jude) and its affiliate sites. Therapy for ALL/LLy is unique; it lasts approximately 2.5 years and can exacerbate COVID-19–related complications (eg, pancreatitis and thrombosis caused by asparaginase and obesity, diabetes, and lymphopenia-related severe viral infection associated with glucocorticoids). Our primary objective was to describe the spectrum of illness and outcomes associated with COVID-19 and chemotherapy administration. As secondary aims, we evaluated the differences in clinical characteristics between patients who developed COVID-19 and those who did not during the study period, as well as the association of demographic and clinical factors with COVID-19 severity. We hypothesized that most patients receiving treatment for ALL/LLy did not have severe disease but experienced treatment interruptions and modifications and that receiving intense phases of chemotherapy and being older at ALL/LLy diagnosis would increase the risk of severe COVID-19.

## Methods

The study was approved by the St Jude institutional review board, and a waiver of consent from the study participants was granted because patients or legal guardians have signed treatment protocol consent which allows for collection of adverse effect data. The study followed the EQUATOR reporting guideline, specifically those for uncontrolled case series.

### Patients

Patients aged 1 to 18 years with newly diagnosed ALL/LLy who received therapy on the St Jude Total XVII protocol (NCT03117751)^11^ between March 30, 2020, and June 20, 2022, were included. Selected characteristics of participants who developed COVID-19 while receiving therapy were compared with those of participants who did not develop COVID-19 during this period. All patients underwent at least weekly screening for SARS-CoV-2 by qualitative polymerase chain reaction (PCR) testing of nasal swab specimens.

### Clinical Course of COVID-19

Detailed data on patients with SARS-CoV-2 infections were obtained from a retrospective review of electronic medical records and the protocol database. Some data were provided by the Pediatric COVID-19 US Registry^12^ for which St Jude in Memphis, Tennessee, serves as the data collection and coordinating center. Registry data were collected via a publicly available electronic survey by using Research Electronic Data Capture, hosted by St Jude.^13^

Information on race and ethnicity, as defined by patients or their legal guardians, was collected. Race categories were African American or Black, White, and Other (American Indian or Alaska Native, Asian, Native Hawaiian or Other Pacific Islander, and not specified); ethnicity categories were Hispanic, not Hispanic, and not specified. This information was collected to establish the baseline demographic characteristics of the study sample to examine any associations with the outcome variables of occurrence of SARS-CoV-2 infection, severity of COVID-19 illness, and occurrence of SARS-CoV-2 reinfection.

COVID-19 acute symptoms and the modifications of chemotherapy were evaluated for 60 days after the COVID-19 diagnosis. Data were collected on the following variables during the 27-month study period: viral clearance, defined as 2 negative PCR tests being documented at least 24 hours apart; specific adverse events of multisystem inflammatory syndrome in children, thromboses, pancreatitis, and death; and vaccination and the development of a SARS-CoV-2 reinfection. Patients were considered to have a SARS-CoV-2 reinfection if they had a new positive test for SARS-CoV-2 after exhibiting viral clearance.

COVID-19 severity was categorized as follows: asymptomatic to paucisymptomatic (no attributable symptoms, rhinorrhea, nasal congestion, or intermittent cough in the absence of fever), mild to moderate (fever, myalgia/fatigue, gastrointestinal symptoms, requirement for supplemental oxygen by nasal cannula only, or hospital admission not meeting criteria for severe illness [eg, febrile neutropenia, diarrhea, or dehydration]), or severe (symptoms of chest pain, shortness of breath, tachypnea, increasing oxygen requirement, lobar pneumonia on imaging, or admission to an intensive care unit).^2,9,14,15^ Mild and moderate illness were grouped together because patients in this population may be hospitalized for very mild symptoms, such as fever, that would not be regarded as serious in other populations.

### Statistical Analysis

Descriptive statistics were used for clinical presentation and demographic variables. Associations between categorical variables were evaluated with χ^2^ or Fisher exact tests, and associations between continuous and categorical variables were evaluated with Wilcoxon or Kruskal-Wallis rank-sum tests. The analyses were not adjusted for multiple comparisons because of the exploratory nature of the study. All statistical analyses were conducted using R statistical software, version 4.1.2 (R Foundation for Statistical Computing), and a predetermined α level of significance of .05 was used.

## Results

### Clinical Course and Factors Associated With Severe COVID-19

Of 308 patients with ALL/LLy who were receiving therapy during the study period, 110 (36%) tested positive for SARS-CoV-2 at a median age of 8.2 (IQR, 5.3-14.5) years. Sixty-eight of these patients (62%) were male, 42 (38%) were female, 24 (22%) were of African American or Black race, 17 (15%) were Hispanic, 89 (81%) had non-Hispanic ethnicity, and 81 (74%) were of White race (Table 1). Compared with those without SARS-CoV-2 infection, patients with SARS-CoV-2 infection were older at the diagnosis of ALL/LLy (median age, 6.7 [IQR, 3.7-12.8] vs 5.3 [IQR, 3.0-10.5] years) and more likely to be of Hispanic or unspecified ethnicity. Most patients had mild to moderate illness (62 [56%]) (Table 2). Seventeen SARS-CoV-2 infections (16%) were detected on asymptomatic screening. Seven patients (6%) met criteria for severe COVID-19, 6 of them presenting before the less virulent Omicron variant became the dominant strain in the US (before December 27, 2021) (Figure; eTable 1 in Supplement 1).

**Table 1.** Comparison of Patients With ALL/LLy Who Did and Did Not Develop SARS-CoV-2 Infection

Characteristic	Patients, No. (%)			*P* value
	All (n = 308)	Without SARS-CoV-2 infection (n = 198)	With SARS-CoV-2 infection (n = 110)	
Age at ALL/LLy diagnosis, median (IQR), y	5.8 (3.1-11.4)	5.3 (3.0-10.5)	6.7 (3.7-12.8)	.01
Sex				
Female	114 (37)	72 (36)	42 (38)	.75
Male	194 (63)	126 (64)	68 (62)	
Race				
African American or Black	51 (17)	27 (14)	24 (22)	.14
White	238 (77)	157 (79)	81 (74)	
Other	19 (6.2)	14 (7.1)^a^	5 (4.5)^b^	
Ethnicity				
Hispanic	37 (12)	20 (10)	17 (15)	.04
Not Hispanic	266 (86)	177 (89)	89 (81)	
Not specified	5 (1.6)	1 (0.5)	4 (3.6)	
ALL/LLy lineage				
B cell	245 (80)	159 (80)	86 (78)	.78
T cell	62 (20)	38 (19)	24 (22)	
MPAL	1 (0.3)	1 (0.5)	0 (0)	
Risk group				
Low risk	143 (46)	94 (47)	49 (45)	.62
Standard/high risk	165 (54)	104 (53)	61 (55)	
Initial WBC count, /μL				
<50 000	243 (79)	155 (78)	88 (80)	.72
≥50 000	65 (21)	43 (22)	22 (20)	
CNS status				
Negative	215 (70)	138 (70)	77 (70)	.96
Positive	93 (30)	60 (30)	33 (30)	

Abbreviations: ALL, acute lymphoblastic leukemia; CNS, central nervous system; LLy, lymphoblastic lymphoma; MPAL, mixed-phenotype acute leukemia; WBC, white blood cell.

SI conversion factor: To convert WBC to  × 10^9^ per microliter, multiply by 0.001.

^a^ American Indian/Alaskan Native (n = 1), Asian (n = 4), multiple race (n = 6) (both White and Black/African American), not specified (n = 3).

^b^ Asian (n = 3), American Indian/Alaskan Native (n = 2).

**Table 2.** Characteristics of Patients With COVID-19 and Association With Illness Severity

Characteristic	No. (%)				*P* value
	All patients (n = 110)	Asymptomatic/paucisymptomatic (n = 41)	Mild/moderate (n = 62)	Severe (n = 7)	
**Baseline features of ALL/LLy**					
Age at diagnosis, median (IQR), y	6.7 (3.7-12.8)	5.9 (3.2-10.7)	6.4 (3.5-12.8)	15.7 (14.2-16.2)	.005
Initial WBC groups, /μL					
<50 000	88 (80)	31 (76)	54 (87)	3 (43)	.02
≥50 000	22 (20)	10 (24)	8 (13)	4 (57)	
CNS status					
Negative	77 (70)	29 (71)	44 (71)	4 (57)	.76
Positive^a^	33 (30)	12 (29)	18 (29)	3 (43)	
Lineage^b^					
B cell	86 (78)	34 (83)	47 (76)	5 (71)	.61
T cell	24 (22)	7 (17)	15 (24)	2 (29)	
Risk group					
Low	49 (45)	21 (51)	27 (44)	1 (14)	.18
Standard/high	61 (55)	20 (49)	35 (56)	6 (86)	
**Demographics**					
Age at COVID-19 diagnosis, median (IQR), y	8.2 (5.3-14.5)	6.8 (4.8-12.5)	8.2 (5.1-14.2)	16.5 (15.2-17.8)	.009
Sex					
Female	42 (38)	18 (44)	22 (35)	2 (29)	.67
Male	68 (62)	23 (56)	40 (65)	5 (71)	
BMI at COVID-19 diagnosis					
<85th percentile	58 (53)	18 (45)	36 (58)	4 (57)	.36
≥85th percentile	52 (47)	23 (56)	26 (42)	3 (43)	
Race					
African American or Black	24 (22)	11 (27)	12 (19)	1 (14)	.26
White	81 (74)	30 (73)	46 (74)	5 (71)	
Other	5 (4.5)	0	4 (6.5)^c^	1 (14)^d^	
Ethnicity					
Hispanic	17 (15)	8 (20)	9 (15)	0	.38
Not Hispanic	89 (81)	30 (73)	52 (84)	7 (100)	
Not specified	4 (3.6)	3 (7.3)	1 (1.6)	0	
**Initial SARS-CoV-2 infection**					
Timing in pandemic^e^					
Pre-Omicron	55 (50)	20 (49)	29 (47)	6 (86)	.20
Post-Omicron	55 (50)	21 (51)	33 (53)	1 (14)	
Phase of ALL/LLy treatment					
Induction to continuation week 19^f^	33 (30)	9 (22)	20 (32)	4 (57)	.14
Continuation week 20-120	77 (70)	32 (78)	42 (68)	3 (43)	
Steroids in the last 14 d					
No	74 (67)	29 (71)	39 (63)	6 (86)	.46
Yes	36 (33)	12 (29)	23 (37)	1 (14)	
Asparaginase in the last 14 d					
No	95 (86)	39 (95)	50 (81)	6 (86)	.09
Yes	15 (14)	2 (4.9)	12 (19)	1 (14)	
WBC at COVID-19 diagnosis, median (IQR) (n = 98) /μL^g^	2.29 (1.44-3.62)	2.50 (1.91-3.68)	2.10 (1.37-3.62)	1.20 (0.82-2.22)	.09
ANC at COVID-19 diagnosis (/μL), median (IQR) (n = 98)^g^	1300 (702-2446)	1200 (730-2415)	1465 (739-2572)	700 (518-1686)	.44
ALC at COVID-19 diagnosis (/μL), median (IQR) (n = 98)^g^	487 (297-758)	600 (347-1040)	470 (289-710)	300 (234-350)	.02
Concurrent respiratory co-infection^h^					
No	94 (85)	35 (85)	55 (89)	4 (57)	.10
Yes	16 (15)	6 (15)	7 (11)	3 (43)	
Chest imaging abnormalities (n = 28)^i^					
No	14 (50)	1 (100)	13 (65)	0 (0)	.006
Yes	14 (50)	0 (0)	7 (35)	7 (100)	
Chemotherapy held/delayed					
No	14 (13)	10 (24)	4 (6.5)	0 (0)	.03
Yes	96 (87)	31 (76)	58 (94)	7 (100)	
Days chemotherapy held, median (IQR)	8 (7-14)	7 (5-11)	9 (7-14)	17 (13-24)	<.001
COVID-19-directed therapy^j^					
No	88 (80)	41 (100)	46 (74)	1 (14)	<.001
Yes	22 (20)	0 (0)	16 (26)	6 (86)	
SARS-CoV-2 reinfection					
No	99 (90)	37 (90)	58 (94)	4 (57)	.03
Yes	11 (10)	4 (9.8)	4 (6.5)	3 (43)	

Abbreviations: ALC, absolute lymphocyte count; ALL, acute lymphoblastic leukemia; ANC, absolute neutrophil count; BMI, body mass index; CNS, central nervous system; LLy, lymphoblastic lymphoma; WBC, white blood cell.

SI conversion factor: To convert ANC, ALC, and WBC to  × 10^9^ per microliter, multiply by 0.001.

^a^ CNS-positive includes CNS 2, CNS 3, and traumatic tap with blasts.

^b^ ALL/LLy: B-ALL: 82 (75%), B-LLy: 4 (3.6%), T-ALL: 17 (15%), T-LLy: 7 (6.4%).

^c^ American Indian/Alaskan Native, n = 2; Asian, n = 2.

^d^ Asian.

^e^ Pre-Omicron: March 30, 2020, to December 26, 2021; post-Omicron: December 27, 2021, to June 20, 2022.

^f^ Induction (n = 1), consolidation (n = 6), blinatumomab (n = 2), continuation weeks 1 to 19 (n = 24).

^g^ These values are from blood counts checked within ±3 days of COVID diagnosis; patients for whom laboratory values were not available within this timeframe were excluded (n = 12).

^h^ Rhinovirus/enterovirus (n = 11), adenovirus (n = 2), parainfluenza 3 (n = 1), human metapneumovirus (n = 1), influenza B (n = 1), and respiratory syncytial virus (n = 1), coronavirus OC43 (n = 1). One patient had coronavirus OC43, rhinovirus/enterovirus, and parainfluenza, and another patient had influenza B and rhinovirus concurrently.

^i^ Chest radiograph: n = 27; chest computed tomography: n = 6; 1 patient with severe illness due to pancreatitis had a chest computed tomography but not a chest radiograph.

^j^ COVID-19–directed therapy included remdesivir (n = 11), intravenous immunoglobulin (n = 9), convalescent plasma (n = 1), casirivimab with imdevimab (n = 1), sotrovimab (n = 1), baricitinib (n = 1), and steroids (n = 5); others (prior to clinical evidence) included hydroxychloroquine (n = 1), azithromycin (n = 3), and ivermectin (n = 1).

**Figure.**
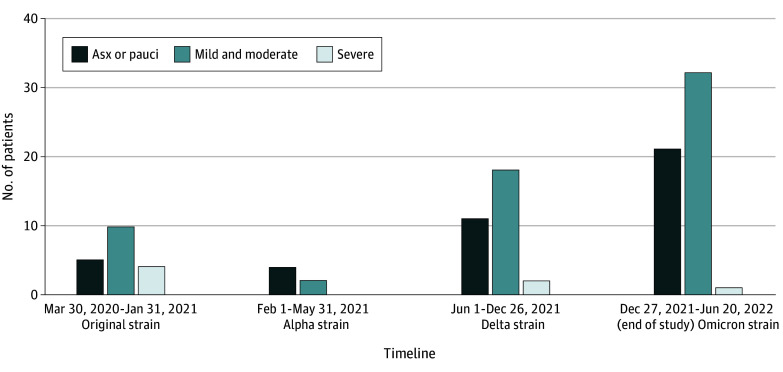
Distribution of the Severity of COVID-19 Symptoms Over the Study Period Data have been categorized by dates based on the waves of the most common circulating variants in the US from the original strain to the end of the study period. Asx or pauci indicates asymptomatic or paucisymptomatic.

Most patients had COVID-19 in the continuation (maintenance) phase of chemotherapy (101 [92%]), especially in the later part of continuation (after week 20) (77 [70%]). The most common symptoms were cough (69 [63%]), fever (52 [47%]), and rhinorrhea (46 [42%]) (eTable 2 in Supplement 1). Thirty-six patients (33%) were admitted to the hospital at the clinician’s discretion for febrile neutropenia (16 [15%]), fever with other symptoms and/or other coinfection (16 [15%]), respiratory distress without fever (3 [2.7%]), or another complication (pancreatitis, 1 [0.9%]). Only 4 patients (3.6%) required intensive care unit admission.

Data on SARS-CoV-2 clearance were missing for 22 patients. Among patients with available data (n = 88), the median time to SARS-CoV-2 clearance was 31.5 days (IQR, 22-47 days; minimum, 11 days; maximum, 126 days). Persistent positivity beyond the 60-day follow-up was noted in 13 of 88 patients (15%). The duration of clearance was not associated with COVID-19 severity.

A chest radiograph was performed for 27 patients (25%) for cough and fever at COVID-19 presentation (n = 15), for persistent cough over the course of the illness (n = 6), or at the onset of respiratory distress (n = 6). All patients with severe COVID-19 had infiltrates, consolidation, and/or pleural effusions (eTable 1 in Supplement 1), whereas patchy infiltrates were seen in 7 of the 20 patients with mild to moderate disease who underwent imaging. Computed tomography of the chest was performed for 6 of the 7 patients with severe COVID-19 and confirmed chest radiograph findings of ground-glass infiltrates in 4 patients and pleural effusions in 2 patients.

Eleven patients (10%) were given remdesivir as COVID-19–directed therapy. Seven of them received a 3-day course to prevent progression to lower respiratory tract infection, whereas 4 received a minimum 5-day course for severe disease. Three patients with severe disease received dexamethasone. No patients received combination tixagevimab and cilgavimab. Anticoagulation use was significantly associated with older age (median age, 14.8 [IQR, 8.3-16.6] vs 7.8 [IQR, 4.8-13.3] years; *P* = .002), being used in an earlier phase of ALL/LLy therapy (induction to continuation week 19) (64% vs 25%; *P* = .005), and with having severe COVID-19 (36% vs 2%; *P* < .001) (eTable 3 in Supplement 1).

Severe COVID-19 was associated with older age at ALL/LLy diagnosis (median age, 15.7 [IQR, 14.2-16.2] years for patients with severe disease vs 6.4 [IQR, 3.5-12.8] and 5.9 [IQR, 3.2-10.7] years for patients with mild/moderate and asymptomatic/paucisymptomatic disease, respectively; *P* = .005), a white blood cell count at ALL/LLy diagnosis of ≥50000/μL (to convert this value to the number ×10^9^ per microliter, multiply by 0.001) (found in 57% of patients with severe disease vs 13% and 24% of patients with mild/moderate and asymptomatic/paucisymptomatic disease, respectively, *P* = .02), older age at COVID-19 diagnosis (median age, 16.5 [IQR, 15.2-17.8] years for patients with severe disease vs 8.2 [IQR, 5.1-14.2] and 6.8 [IQR, 4.8-12.5] years for patients with mild/moderate and asymptomatic/paucisymptomatic disease, respectively; *P* = .009), a lower absolute lymphocyte count at COVID-19 diagnosis (median, 300/μL [IQR, 234-350] for patients with severe disease vs 470/μL [IQR, 289-710] and 600/μL [IQR, 347-1040] for patients with mild/moderate and asymptomatic/paucisymptomatic disease, respectively; *P* = .02 [to convert these values to the number ×10^9^ per microliter, multiply by 0.001]), the presence of abnormalities on chest imaging during COVID-19 illness (present in 100% of patients with severe disease vs 35% and 0% of patients with mild/moderate and asymptomatic/paucisymptomatic disease, respectively; *P* = .006), receiving COVID-19–directed therapy (received by 86% of patients with severe disease vs 26% and 0% of patients with mild/moderate and asymptomatic/paucisymptomatic disease, respectively; *P* < .001), and developing SARS-CoV-2 reinfection (which occurred in 43% of patients with severe disease vs 6.5% and 9.8% of patients with mild/moderate and asymptomatic/paucisymptomatic disease, respectively; *P* = .03) (Table 2).

No patients required mechanical ventilation, developed multisystem inflammatory syndrome in children, or died in the observation period. Sixteen of 110 patients received asparaginase within 21 days of their COVID-19 diagnosis. In terms of serious complications, 2 children developed thrombosis and 2 had pancreatitis (1 of the latter also had thrombosis) during the study period. One patient presented with pancreatitis at the time of COVID-19 diagnosis and subsequently developed lower-extremity deep venous thrombosis and pulmonary embolism 11 days after the COVID-19 diagnosis. Another patient experienced seizures 49 days after the COVID-19 diagnosis (3 weeks after SARS-CoV-2 clearance), and imaging revealed cerebral venous sinus thrombosis requiring thrombectomy and anticoagulation. This patient was receiving prophylactic enoxaparin throughout their COVID-19 illness; however, this was discontinued upon documentation of viral clearance. These patients with serious thrombotic events had received pegaspargase; the first patient 3 weeks and the second patient 1 week before their COVID-19 diagnosis. A remaining patient developed pancreatitis 139 days after COVID-19, having received a dose of pegaspargase 2 days beforehand.

### Chemotherapy Outcomes

Chemotherapy was held until clinical improvement and/or completion of antiviral therapy in 96 of the 110 patients (87%) for a median of 8 (IQR, 7-14) days. Chemotherapy was held for all patients in the induction and consolidation treatment phases and for most patients in the continuation/maintenance phase (eTable 4 in Supplement 1). Chemotherapy was held more often and for longer times in patients with severe disease, compared with other patients (Table 2). However, these modifications were also made in 76% of the asymptomatic/paucisymptomatic patients. There was no significant difference in the frequency of chemotherapy holds in the pre-Omicron vs post-Omicron periods (50 [91%] vs 45 [82%]; *P* = .09). However, the median duration of chemotherapy hold was longer in the pre-Omicron period (12 [IQR, 7-14] vs 7 [IQR, 6-10] days; *P* < .001).

### SARS-CoV-2 Reinfection

SARS-CoV-2 reinfection (second COVID-19) occurred in 11 patients (10%) and was associated with older age at ALL/LLy diagnosis and at first SARS-CoV-2 infection, severe illness with the first infection, and receiving standard/high-risk vs low-risk ALL/LLy therapy (Table 2 and Table 3). The median interval between initial and second COVID-19 episodes was 8.8 (range, 2.5-19.3) months, and all patients had negative SARS-CoV-2 PCR tests before the second infection. No patients had severe disease with their second episode, and only 1 patient received a 3-day course of remdesivir (eTable 5 in Supplement 1). Receiving remdesivir with the first infection did not affect the development of a second infection. Chemotherapy was held for 9 of the 11 patients during the second episode (median, 10 [IQR, 7-14] days), resulting in the median total chemotherapy interruption being significantly longer for this subpopulation (median, 22 [IQR, 20-27] days) than for patients who had a single episode (median, 8 [IQR, 7-14] days) (Table 3).

**Table 3.** Comparative Characteristics of Patients With and Without SARS-CoV-2 Reinfection

Characteristic at initial SARS-CoV-2 infection	Patients, No. (%)		*P* value
	Without SARS-CoV-2 reinfection (n = 99)	With SARS-CoV-2 reinfection (n = 11)	
Age at ALL/LLy diagnosis, median (IQR), y	6.2 (3.4-12.5)	12.8 (9.8-15.0)	.03
WBC count at ALL/LLy diagnosis, /μL			
<50 000	80 (81)	8 (73)	.47
≥50 000	19 (19)	3 (27)	
CNS status at ALL/LLy diagnosis			
Negative	69 (70)	8 (73)	>.99
Positive	30 (30)	3 (27)	
ALL/LLy lineage			
B cell	79 (80)	7 (64)	.25
T cell	20 (20)	4 (36)	
Risk group			
Low risk	48 (48)	1 (9.1)	.02
Standard/high risk	51 (52)	10 (91)	
Age at COVID-19 diagnosis, median (IQR), y	7.8 (5.0-13.6)	14.2 (11.4-15.6)	.04
BMI at COVID-19 diagnosis, median (IQR)	18.8 (16.7-22.3)	19.1 (16.8-21.0)	.92
Sex			
Female	40 (40)	2 (18)	.20
Male	59 (60)	9 (82)	
Ethnicity			
Hispanic	16 (16)	1 (9.1)	>.99
Not Hispanic	79 (80)	10 (91)	
Not specified	4 (4.0)	0	
Race			
African American or Black	22 (22)	2 (18)	>.99
White	72 (73)	9 (82)	
Other^a^	5 (5.1)	0	
Phase of ALL/LLy treatment			
Induction to continuation week 19	27 (27)	6 (55)	.08
Continuation week 20-120	72 (73)	5 (45)	
Days chemotherapy held, median (IQR)	8 (7-14)	12 (8-18)^b^	.05
Total days of chemotherapy hold (first and second episode if applicable), median (IQR)	8 (7-14)	22 (20-27)	<.001
COVID-19–directed therapy with first SARS-CoV-2 infection	20 (20)	2 (18)	>.99

Abbreviations: ALL, acute lymphoblastic leukemia; BMI, body mass index (calculated as weight in kilograms divided by height in meters squared); CNS, central nervous system; LLy, lymphoblastic lymphoma; WBC, white blood cell.

SI conversion factor: To convert WBC to  × 10^9^ per microliter, multiply by 0.001.

^a^ Asian (n = 3), American Indian/Alaskan Native (n = 2).

^b^ First episode only.

### SARS-CoV-2 Vaccination

Fifty-one of 110 patients (46%) had SARS-CoV-2 infection before vaccines became available or were indicated for their age. Of the 59 patients who were eligible for the vaccine before their first infection, 19 (32%) received 1 or more doses (eFigure in Supplement 1). Five had parental refusal and 2 had medical contraindication documented as reasons for not being vaccinated. No documentation was available for the remaining 33 patients. Fifteen patients who did not receive the vaccine before their first SARS-CoV-2 infection for various reasons received 1 or more doses after the first or second SARS-CoV-2 infection. Of the 7 patients with severe COVID-19, 4 were ineligible for vaccination before their infection and 3 were eligible, with 2 of the latter having vaccination data, but none received any vaccine doses before their first infection.

## Discussion

During our observation period, SARS-CoV-2 infections occurred in 110 of 308 patients (36%), a rate substantially higher than the cumulative incidence of 18% in our general pediatric population^16^; this is probably reflective of routine screening (at least weekly) in the patients in this study. The higher incidence in older children might be explained by the risk of exposure to SARS-CoV-2 during school and social activities, whereas the higher risk in Hispanic children is not well understood but is consistent with the findings of other studies.^10^ Severe COVID-19 occurred in only 7 of 110 patients (6%), a low incidence consistent with findings in European countries.^7,8,17^ It has been hypothesized that immunosuppression decreases the inflammatory response to COVID-19, thus limiting disease-related damage and contributing to a lower severity than anticipated in this patient population.^18^ Only 4 of the patients (4%) in this study required intensive care unit admission; a higher incidence (11%) was reported for another US cohort of patients with hematologic malignancies, but that study included patients with relapsed or refractory disease.^10^

Consistent with other studies,^2,9,10^ most of the patients with severe COVID-19 in our study were adolescents. Age is a well-documented risk factor for severe COVID-19.^19^ Within the pediatric population, infants and patients aged 15 to 19 years have more severe disease compared with patients aged 5 to 9 years. Several explanations for severe COVID-19 in adolescents have been proposed.^3,20^ The more mature binding ability of the SARS-CoV-2 viral receptor (angiotensin-converting enzyme 2), the fewer restrictions on activities that increase exposure to SARS-CoV-2, and the higher incidences of adverse effects and comorbidities resulting from more intensive chemotherapy (eg, lower absolute lymphocyte count, obesity, and diabetes) in older patients than in younger patients may play roles. The associations of severe disease with lower absolute lymphocyte count at COVID-19 diagnosis^2^ and with chest imaging abnormalities were expected.

SARS-CoV-2 reinfection was seen in 11 patients (10%). It was associated with older age at ALL/LLy diagnosis and at first SARS-CoV-2 infection, with severe illness with the first infection, and with receiving standard/high-risk vs low-risk ALL/LLy therapy. As all of these patients had 2 negative SARS-CoV-2 PCR tests before the second infection and the median interval between the initial and second SARS-CoV-2 infections was 8.8 months, it is unlikely that the apparent reinfection was due to residual initial infection.

There is no standardized approach to chemotherapy modification, which is commonly reported for patients with mild to moderate COVID-19 illness.^8,10,17,21,22^ Chemotherapy can be continued without adverse events, especially for patients in the lower-intensity treatment phases.^17,23^ All patients with severe COVID-19 had chemotherapy held or delayed, as did 94% of the patients with mild to moderate infection and 76% of those who were asymptomatic or paucisymptomatic. The decisions were, rather, clinician dependent because of concern over worsening COVID-19 symptoms; however, there are no clear data showing the benefit of this practice, especially in asymptomatic or paucisymptomatic patients. Most SARS-CoV-2 infections in the patients in this study were seen in the less intensive continuation (maintenance) phase, probably reflecting greater community exposure in this phase, when most chemotherapy is administered in the outpatient setting. In the post-Omicron period, chemotherapy interruption was shorter, possibly because cases were less severe and because clinicians had accumulated more experience. Regardless, such modifications and delays should be minimized or a lower-intensity regimen should be given, especially during the early phase of therapy. We made up the missed chemotherapy if a modification was made within 8 months after the ALL/LLy diagnosis. Receiving less than 95% of the prescribed mercaptopurine doses because of nonadherence in the maintenance phase of ALL treatment increases the risk of relapse 2.7-fold.^24^ As the events of ALL can occur up to 5 years after diagnosis, we could not evaluate the ALL/LLy outcomes in this study because of the short follow-up. Therefore, there is a need for studies with longer follow-up periods and consideration of COVID-19 and/or chemotherapy interruption. COVID-19 can affect survival outcomes of patients with ALL/LLy through treatment interruptions and COVID-19–associated adverse effects, although treatment interruption and modifications are also common in response to toxicities other than COVID-19. Alternatively, outcomes can be compared between patients who had COVID-19 and those who did not to examine the influence of the disease by itself.

COVID-19–directed therapy and anticoagulation were considered based on disease severity as part of the recommended supportive care.^23,25^ Coagulopathy is a known adverse effect of asparaginase, adding to the risk of thrombosis from COVID-19, as seen in 2 of the patients in this study. Even with prophylactic anticoagulation, thrombotic complications should be considered in patients with suggestive symptoms.^23^

### Limitations

There are limitations to interpreting our study because of its retrospective observational design and the small sample size. The study covered time periods in which different SARS-CoV-2 variants (pre-Omicron strains and Omicron) with different clinical pictures predominated and periods before and after vaccinations and immunity against the virus were developed, which can make it difficult to draw firm conclusions. Additionally, our follow-up was limited to the study period; therefore, the true incidence of delayed adverse events (thromboses, pancreatitis, and encephalopathy) and ALL/LLy outcomes cannot be determined from the data.

Vaccination against SARS-CoV-2 has been proved to reduce the severity of COVID-19 illness.^26^ However, the limited sample size, lack of information on prior vaccination, and variability in the number of doses and the time from the last dose to infection has impeded further statistical analysis. This may partly reflect overall low rates of vaccine uptake in children, particularly in the southwest US.^27^ Severe COVID-19 was rare in our cohort, but none of the patients with severe disease and available vaccination data had received a dose before becoming infected.

## Conclusions

In this case series of pediatric patients with ALL/LLy and COVID-19, severe COVID-19 was rare, but chemotherapy administration was affected. The influence of COVID-19 on outcomes should be determined by long-term studies.

## Notes

Supplement 1. **eTable 1**. Characteristics of Patients Categorized as Having Severe COVID-19 **eTable 2.** Symptoms of Patients With COVID-19 **eTable 3.** Use of Anticoagulation in Patients With COVID-19 **eTable 4.** Chemotherapy Modifications With First SARS-Cov-2 Infection **eTable 5.** Comparison of Initial and Second SARS-Cov-2 Infections **eFigure.** Timeline of SARS-CoV-2 Vaccination and SARS-COV-2 Infection

Supplement 2.
Data Sharing Statement
