# Supplementary material for: COVID-19 in Pediatric Patients With Acute Lymphoblastic Leukemia or Lymphoma
Source: JAMA Netw Open. 2024 Feb 16;7(2):e2355727. doi: 10.1001/jamanetworkopen.2023.55727 (PMC10873761; doi:10.1001/jamanetworkopen.2023.55727)
Supplement: Supplement 1. — eTable 1. Characteristics of Patients Categorized as Having Severe COVID-19 eTable 2. Symptoms of Patients With COVID-19 eTable 3. Use of Anticoagulation in Patients With COVID-19 eTable 4. Chemotherapy Modifications With First SARS-Cov-2 Infection eTable 5. Comparison of Initial and Second SARS-Cov-2 Infections eFigure. Timeline of SARS-CoV-2 Vaccination and SARS-COV-2 Infection [file jamanetwopen-e2355727-s001.pdf]

Supplementary Online Content

Hashmi SK, Bodea J, Patni T, et al. COVID-19 in pediatric patients with acute lymphoblastic leukemia/lymphoma. *JAMA Netw Open*. 2024;7(2):e2355727. doi:10.1001/jamanetworkopen.2023.55727

eTable 1. Characteristics of Patients Categorized as Having Severe COVID-19

eTable 2. Symptoms of Patients With COVID-19

eTable 3. Use of Anticoagulation in Patients With COVID-19

eTable 4. Chemotherapy Modifications With First SARS-Cov-2 Infection

eTable 5. Comparison of Initial and Second SARS-Cov-2 Infections

eFigure. Timeline of SARS-CoV-2 Vaccination and SARS-COV-2 Infection

This supplementary material has been provided by the authors to give readers additional information about their work.

© 2024 Hashmi SK et al. *JAMA Network Open.*

eTable 1. Characteristics of patients categorized as having severe COVID-19

		Characteristics by individual case						
**Characteristic**	***N* = 7**<br>**No. (%)**	**ID #1**	**ID #20**	**ID #25**	**ID #42**	**ID #47**	**ID #77**	**ID #88**
Age at ALL/LLy diagnosis in years, median (IQR)	15.70<br>(13.84–16.50)	18.16	16.50	14.63	15.81	15.70	6.74	13.84
Age at COVID-19 diagnosis in years, median (IQR)	16.52<br>(14.18–18.99)	20.29	18.99	16.52	16.57	16.28	6.76	14.18
Sex<br>Female<br>Male	 2 (28.6)<br>5 (71.4)	Female	Male	Male	Female	Male	Male	Male
Race<br>White<br>Black<br>Other[a]	 5 (71.4)<br>1 (14.3)<br>1 (14.3)[a]	Black	White	White	White	White	Other	White
Ethnicity<br>Hispanic<br>Non-Hispanic	 0 (0)<br>7 (100)	Non-Hispanic	Non-Hispanic	Non-Hispanic	Non-Hispanic	Non-Hispanic	Non-Hispanic	Non-Hispanic
Immunophenotype<br>B cell<br>T cell	 5 (71.4)<br>2 (28.6)	B-ALL	B-ALL	B-ALL	B-ALL	B-ALL	T-ALL	T-ALL
Risk group<br>Low risk<br>Standard/high risk	 1 (14.3)<br>6 (85.7)	SR/HR	SR/HR	SR/HR	SR/HR	LR	SR/HR	SR/HR
WBC at ALL/LLy diagnosis, (× 10^3/μL)[b], median (IQR)<br><50<br>≥50	51.1 (16.4–94.5)<br>4 (57.1)<br>3 (42.9)	199	51.1	77.3	16.4	47.6	94.5	13.8
**Characteristic**	***N* = 7**	**ID #1**	**ID #20**	**ID #25**	**ID #42**	**ID #47**	**ID #77**	**ID #88**

© 2024 Hashmi SK et al. *JAMA Network Open.*

	No. (%)							
CNS status<br>CNS 1<br>CNS 2<br>CNS 3<br>Traumatic LP with blasts	 4 (57.1)<br>3 (42.9)<br>0 (0)<br>0 (0)	CNS 2	CNS 2	CNS 1	CNS 1	CNS 1	CNS 2	CNS 1
Timing in pandemic<br>Pre-Omicron<br>Post-Omicron	 6 (85.7)<br>1 (14.3)	Pre-Omicron	Post-Omicron	Pre-Omicron	Pre-Omicron	Pre-Omicron	Pre-Omicron	Pre-Omicron
Phase of therapy<br>Induction until continuation week 19<br>Continuation week 20 until week 120 (end of therapy)	 4 (57.1)<br>3 (42.9)	Continuation week 90	Continuation week 95	Continuation week 75	Continuation week 19	Continuation week 12	Induction day 7	Consolidation day 43
Steroids in last 14 days	1 (14.3)	N	N	N	Y	N	N	N
Asparaginase in last 14 days	1 (14.3)	N	N	N	Y	N	N	N
Respiratory symptoms	6 (85.7)	Y	Y	Y	Y	N	Y	Y
Gastrointestinal symptoms	5 (71.4)	N	Y	N	Y	Y	Y	Y
Thrombosis	1 (14.3)	N	N	N	N	Y[c]	N	N
Pancreatitis	1 (14.3)	N	N	N	N	Y	N	N
Chest X-ray findings								
Patchy infiltrates	4 (66.7)[d]	Y	Y	N	Y	Not done[c]	N	Y
Focal consolidation	2 (33.3)[d]	N	N	Y	N	Not done[c]	Y	N
Pleural effusion	2 (33.3)[d]	Y	N	N	Y	Not done[c]	N	N
Chest CT findings								
Ground-glass infiltrates/ consolidation	5 (100)[d]	Y	Y	Not done	Y	Y[c]	Y	Not done
Pleural effusions	2 (40)[d]	Y	N	Not done	Y	N[c]	N	Not done
Oxygen requirement	4 (57.1)	N	Y (NC)	N	Y (NC)	N	Y (HFNC)	Y (NC)
ICU admission	4 (57.1)	Y	N	N	N	Y	Y	Y
**Characteristic**	***N* = 7**	**ID #1**	**ID #20**	**ID #25**	**ID #42**	**ID #47**	**ID #77**	**ID #88**

© 2024 Hashmi SK et al. *JAMA Network Open.*

	No. (%)							
WBC at COVID-19 diagnosis (× 10^3/μL)[b], median (IQR)	1.20 (0.74–2.64)	0.9	3.3	1.8	0.74	1.2	2.64	0.6
ANC at COVID-19 diagnosis (/μL)[b], median (IQR)	700 (500–2373)	500	2838	1000	536	700	2373	300
<500	1 (14.3)							
ALC at COVID-19 diagnosis (/μL)[b], median (IQR)	300 (198–400)	300	198	400	138	400	267	300
<200	2 (28.6)							
Chemotherapy interrupted	7 (100)	Y	Y	Y	Y	Y	Y	Y
Number of days chemotherapy was held, median (IQR)	17 (12–24)	14	17	8	34	23	24	12
Chemotherapy omitted	4 (57.1)	Y	Y	Y	N	Y	N	N
Chemotherapy made up	3 (42.9)	N	N	N	Y	N	Y	Y
Use of anticoagulation	5 (71.4)	N	Y	N	Y	Y	Y	Y
COVID-19–directed therapy	5 (71.4)	Y	Y	N	Y	N	Y	Y
Remdesivir	4 (57.1)	N	Y (5 days)	N	Y (5 days)	N	Y (12 days)	Y (5 days)
Corticosteroids	3 (42.9)	N	N	N	Y	N	Y	Y
Hydroxychloroquine[e]	1 (14.3)	Y	N	N	N	N	N	N
Convalescent plasma	1 (14.3)	N	N	N	N	N	Y	N
IVIG	1 (14.3)	N	Y	N	N	N	N	N
Baricitinib	1 (14.3)	N	N	N	N	N	Y	N
Viral clearance, median (IQR), days	49 (23.0–55.5)	60	N/A	N/A	49	27	51	19
Second SARS-CoV-2 infection	3 (42.9)	N	N	N	Y	Y	N	Y
Second COVID-19 severity score[f]		N/A	N/A	N/A	2	1	N/A	2

© 2024 Hashmi SK et al. *JAMA Network Open.*

Abbreviations: ALL, acute lymphoblastic leukemia; LLy, lymphoblastic lymphoma; IQR, interquartile range; COVID-19, coronavirus disease 2019; EOI, end of induction; LR: low risk; SR/HR: standard risk/high risk; WBC, white blood cell; CNS, central nervous system; LP, lumbar puncture; CT, computed tomography; NC, nasal cannula; HFNC, high-flow nasal cannula; ICU, intensive care unit; ANC, absolute neutrophil count; ALC, absolute lymphocyte count.

Footnotes:
[a] Race Other: Asian
[b] SI conversion factor: To convert white blood cell count, absolute neutrophil count, and absolute lymphocyte count to 10^9/L, multiply by 1.0.
[c] Patient #47: The patient was admitted initially with pancreatitis and had an abdominal CT; he also had upper respiratory symptoms and tested positive for SARS-CoV-2, rhino/enterovirus, and influenza B. The patient did not undergo CXR because of the lack of additional respiratory symptoms at the time. Subsequently, he had a 1-week admission for febrile neutropenia, then at approximately 11 days post COVID-19 diagnosis, he developed lower-extremity DVT after presenting with pain and swelling in his right leg. A CT angiogram was obtained at this time, as the patient had also complained of chest pain; this showed a right saddle pulmonary embolus. A new nodular opacity was seen in the right lower lobe (infarct versus pneumonia) on this chest CT. The patient received treatment-dosing of rivaroxaban for 3 months and prophylactic dosing for an additional 9 months until resolution of all thromboses.
[d] Denominator adjusted for number of patients with available data (n = 6 for chest X-ray findings, n = 5 for chest CT findings).
[e] Prior to the availability of clinical evidence.
[f] Severity score: 1: asymptomatic to pauci-symptomatic; 2: mild to moderate

© 2024 Hashmi SK et al. *JAMA Network Open.*

eTable 2. Symptoms of patients with COVID-19

Symptom	*N* = 110<br>No. (%)
Cough	69 (63)
Fever	52 (47)
Rhinorrhea	46 (42)
Fatigue	24 (22)
Decreased appetite	18 (16)
Headache	15 (14)
Vomiting	14 (13)
Nausea	13 (12)
Diarrhea	12 (11)
Ageusia	12 (11)
Abdominal pain	6 (5.5)
Anosmia	6 (5.5)
Myalgia	6 (5.5)
Pharyngitis	6 (5.5)

Abbreviations: COVID-19, coronavirus disease 2019

© 2024 Hashmi SK et al. *JAMA Network Open.*

eTable 3. Use of Anticoagulation in patients with COVID-19

Characteristics	Overall, *N* = 110 No. (%)	No anticoagulation, *N* = 96 No. (%)	Anticoagulation, *N* = 14 No. (%)	*P* value
Age at COVID-19 diagnosis in years, median (IQR)	8.2 (5.3 –14.3)	7.8 (4.8 –13.3)	14.8 (8.3 –16.6)	.002
Phase of ALL/LLy treatment				.005
Induction till Continuation week 19	33 (30)	24 (25)	9 (64)	
Continuation week 20 onwards	77 (70)	72 (75)	5 (36)	
COVID-19 severity				<.001
Asymptomatic to pauci-symptomatic	41 (37)	39 (41)	2 (14)	
Mild to moderate	62 (56)	55 (57)	7 (50)	
Severe	7 (6.4)	2 (2.1)	5 (36)	

Abbreviations: IQR, interquartile range; COVID-19, coronavirus disease 2019; ALL, acute lymphoblastic leukemia; LLy, lymphoblastic lymphoma.

© 2024 Hashmi SK et al. *JAMA Network Open.*

eTable 4. Chemotherapy modifications with first SARS-CoV-2 infection

Phase of protocol therapy (No. of patients per phase)	No. of patients with chemotherapy holds per phase of chemotherapy (%)	Median duration of chemotherapy hold in days (minimum–maximum)
Induction (*N* = 1)	1 (100)	24
Consolidation (*N* = 6)	6 (100)	10.5 (8–12)
Continuation weeks 1–19[a] (*N* = 26)	24 (92)	8 (0–34)
Continuation weeks 20–49 (*N* = 27)	22 (82)	7 (0–21)
Continuation weeks 50–120 (*N* = 50)	43 (86)	10 (0–28)

Footnotes:
[a]Two patients were receiving immunotherapy with blinatumomab before proceeding to the continuation phase of treatment. One patient was on the last day of their course of blinatumomab, and initiation of continuation week 1 (VCR/Dex/Dox/asparaginase/6MP) was delayed by 1 week. The other patient was scheduled to start blinatumomab at COVID-19 diagnosis; blinatumomab was delayed, and interim therapy with oral mercaptopurine/methotrexate was started 5 days after COVID-19 diagnosis and continued for 2 weeks.

© 2024 Hashmi SK et al. *JAMA Network Open.*

eTable 5. Comparison of initial and second SARS-CoV-2 infections

Patient ID	ALL/LLy Immuno-phenotype	Age at COVID-19 (years)		COVID-19 disease severity[a]		Phase of ALL/LLy treatment		Duration of chemotherapy hold (days)		Remdesivir		Time between initial and second COVID-19 (months)[b]
		Initial infection	Second infection	Initial infection	Second infection	Initial infection	Second infection	Initial infection[c]	Second infection[d]	Initial infection	Second infection	
8	B-ALL	15.00	15.23	1	1	Continuation week 100	Continuation week 110	7	0	No	No	2.46
19	T-ALL	12.94	13.28	1	2	Continuation week 95	Continuation week 111	16	7	No	No	4.04
34	B-ALL	2.48	3.96	2	1	Continuation week 8	Continuation week 69	8	14	No	No	19.25
39	B-ALL	17.82	18.37	1	2	Continuation week 51	Continuation week 77	12	10	No	Yes	6.50
42	B-ALL	16.57	17.78	3	2	Continuation week 19	Continuation week 73	34	7	Yes	No	15.14
47[e]	B-ALL	16.28	17.46	3	1	Continuation week 12	Continuation week 62	23	7	No	No	14.79
58	B-ALL	9.80	10.50	1	2	Continuation week 29	Continuation week 64	7	13	No	No	8.82
61	T-ALL	5.65	6.75	2	1	Continuation week 6	Continuation week 60	7	7	No	No	14.00
65	T-ALL	7.14	15.63	2	2	Continuation week 3	Continuation week 40	14	14	No	No	9.89
78	B-ALL	13.53	13.88	2	1	Continuation week 21	Continuation week 36	21	0	No	No	4.04
88[e]	T-ALL	14.18	14.68	3	2	Consolidation Day 43	Continuation week 20	12	14	Yes	No	6.00

Abbreviations: SARS-CoV-2, severe acute respiratory syndrome coronavirus 2; COVID-19, coronavirus disease 2019; ALL, acute lymphoblastic leukemia; LLy, lymphoblastic lymphoma.

Footnotes:
[a]COVID-19 disease severity: 1 = asymptomatic to pauci-symptomatic, 2 = mild to moderate, 3 = severe; [b]median (IQR) (months) = 8.82 (4.04-14.79); [c]median (IQR) (days) = 12 (8-18); [d]median (IQR) (days) = 10 (7-14); [e]SARS-CoV-2 vaccination: Patient 47 received 2 doses at 4.9 and 5.6 months before the second infection (none before the first infection); Patient 88 received 1 dose 4 months after the second infection but none before the first or second infections.

© 2024 Hashmi SK et al. *JAMA Network Open.*

eFigure. Timeline of SARS-CoV-2 vaccination and SARS-COV-2 infection

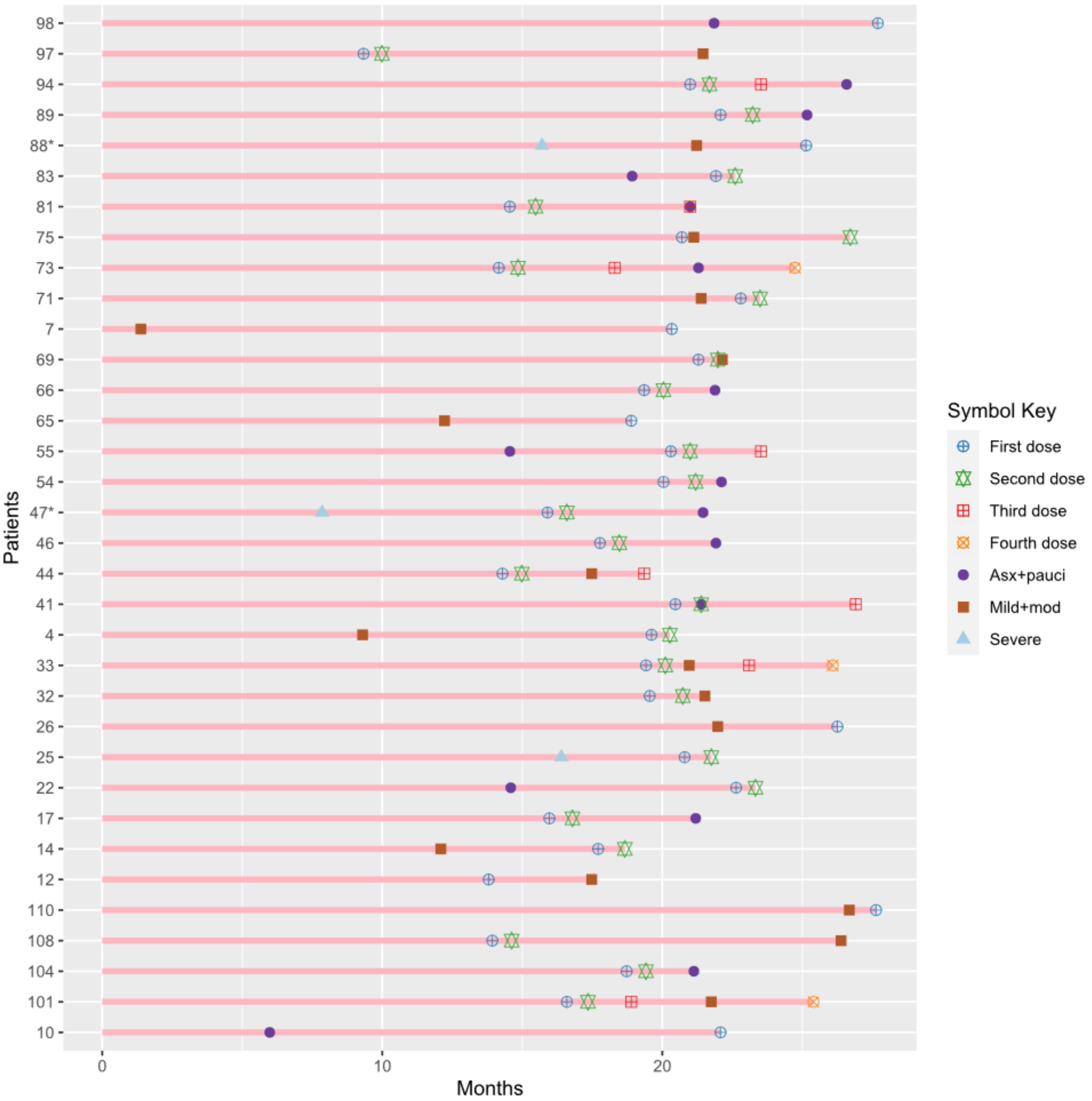

Doses of vaccination and severity of infection for individual patients are denoted by symbols as specified in the symbol key. Nineteen patients (ID nos. 12, 17, 32, 33, 41, 44, 46, 54, 66, 69, 73, 75, 81, 89, 94, 97, 101, 104, and 108) were eligible for and received one or more doses of a SARS-CoV-2 vaccine before their first infection. Three patients (ID nos. 7, 10, and 47) had their first SARS-CoV-2 infection before SARS-CoV-2 vaccines became available (i.e., before December 2020) and 5 patients (ID nos. 4, 14, 22, 55, and 98) had their first infection before SARS-CoV-2 vaccines became available for their age group. Seven patients (ID nos. 25, 26, 65, 71, 83, 88, and 110) were eligible for vaccination before their first infection but did not receive a SARS-CoV-2 vaccine until after their first infection.

*Patients who developed a SARS-CoV-2 reinfection are marked with an asterisk.

Abbreviations: Asx, asymptomatic; pauci, pauci-symptomatic; mod, moderate.

© 2024 Hashmi SK et al. *JAMA Network Open.*
