# Supplementary material for: COVID-19 in Pediatric Patients With Acute Lymphoblastic Leukemia or Lymphoma
Source: JAMA Netw Open. 2024 Feb 16;7(2):e2355727. doi: 10.1001/jamanetworkopen.2023.55727 (PMC10873761; doi:10.1001/jamanetworkopen.2023.55727)
Supplement: Supplement 2. — Data Sharing Statement [file jamanetwopen-e2355727-s002.pdf]

Data Sharing Statement

Hashmi. COVID-19 in Pediatric Patients With Acute Lymphoblastic Leukemia or Lymphoma. *JAMA Netw Open*. Published February 16, 2024. doi:10.1001/jamanetworkopen.2023.55727

Data
Data available: No

Additional Information
Explanation for why data not available: The data that support the findings of this study are available from the corresponding author upon reasonable request.
